# Genome-wide association study of resistance to anthracnose in pepper (*Capsicum chinense*) germplasm

**DOI:** 10.1186/s12870-023-04388-4

**Published:** 2023-08-10

**Authors:** Nayoung Ro, Mesfin Haile, Onsook Hur, Ho-Cheol Ko, Jung-Yoon Yi, Hee-Jong Woo, Yu-Mi Choi, Juhee Rhee, Yong-Jik Lee, Dong-Am Kim, Jae-Wang Do, Geon Woo Kim, Jin-Kyung Kwon, Byoung-Cheorl Kang

**Affiliations:** 1 National Agrobiodiversity Center, National Institute of Agricultural Sciences, Rural Development Administration, Jeonju, Republic of Korea; 2 HANA SEED, Anseong-si, Republic of Korea; 3 Pepper & Breeding Institute, Gimje-si, Republic of Korea; 4 Department of Agriculture, Forestry and Bioresources, Research Institute of Agriculture and Life Sciences, Plant Genomics and Breeding Institute, College of Agriculture and Life Sciences, Seoul National University, Seoul, Republic of Korea

**Keywords:** Anthracnose, *Capsicum chinense*, *Colletotrichum* spp., GWAS, SNPs

## Abstract

**Background:**

Anthracnose is a fungal disease caused by *Colletotrichum* spp. that has a significant impact on worldwide pepper production. *Colletotrichum scovillei* is the most common pathogenic anthracnose-causing species in the Republic of Korea.

**Results:**

The resistances of 197 pepper (*Capsicum chinense*) accessions deposited in Korea’s National Agrobiodiversity Center were evaluated for their response against the virulent pathogens *Colletotrichum acutatum* isolate ‘KSCa-1’ and *C. scovillei* isolate ‘Hana’) in the field and in vitro methods for three consecutive years (2018 to 2020). The severity of the disease was recorded and compared between inoculation methods. Six phenotypically resistant pepper accessions were selected based on three years of disease data. All of the selected resistant pepper accessions outperformed the control resistant pepper in terms of resistance (PI 594,137). A genome-wide association study (GWAS) was carried out to identify single nucleotide polymorphisms (SNPs) associated with anthracnose resistance. An association analysis was performed using 53,518 SNPs and the disease score of the 2020 field and in vitro experiment results. Both field and in vitro experiments revealed 25 and 32 significantly associated SNPs, respectively. These SNPs were found on all chromosomes except Ch06 and Ch07 in the field experiment, whereas in the in vitro experiment they were found on all chromosomes except Ch04 and Ch11.

**Conclusion:**

In this study, six resistant *C. chinense* accessions were selected. Additionally, in this study, significantly associated SNPs were found in a gene that codes for a protein kinase receptor, such as serine/threonine-protein kinase, and other genes that are known to be involved in disease resistance. This may strengthen the role of these genes in the development of anthracnose resistance in *Capsicum* spp. As a result, the SNPs discovered to be strongly linked in this study can be used to identify a potential marker for selecting pepper material resistant to anthracnose, which will assist in the development of resistant varieties.

**Supplementary Information:**

The online version contains supplementary material available at 10.1186/s12870-023-04388-4.

## Introduction

Pepper (*Capsicum* spp.) is one of the most popular and widely grown horticultural crops worldwide due to its variety of products, uses, and forms of consumption [1]. It is native to the tropical areas of South and Central America and has around 38 described species with various morphological differences, mainly noticed in the fruits and related to size, shape, color, and level of pungency [2]. However, of those, only five species are considered domesticated plants: *Capsicum annuum* L., *Capsicum chinense* Jacq., *Capsicum baccatum* L. (var. pendulum), *Capsicum frutescens* L., and *Capsicum pubescens* Ruiz et Pav [3]. *C. chinense* (Habanero pepper) is diploid (2n = 24) and is predominantly self-pollinated. However, cross-pollination plays an important role in increasing the number and quality of fruits as well as facilitating higher seed production [4].

Although *Capsicum* spp. have rich nutritional and economic value, their production has been seriously hampered by several pests and diseases [5]. Of these, *Colletotrichum* is a large genus that includes many important species and prevalent fungal pathogens that cause various tropical and subtropical fruit and vegetable diseases [5]. The genus *Colletotrichum* was ranked as the world’s eighth-most important phytopathogenic fungus [6]. There are several *Colletotrichum* species that cause pepper anthracnose, including *Colletotrichum acutatum* (teleomorph *Glomerella acutata*), *Colletotrichum capsici* (synonym *Colletotrichum dematium*), *Colletotrichum coccodes, Colletotrichum gloeosporioides* (teleomorph *Glomerella cingulata*), and [7]. *C. acutatum* and *C. gloeosporioides* are the most damaging and widely distributed anthracnose disease-causing species [8, 9].

Anthracnose of pepper (*Capsicum* spp.) has become a major limiting factor in pepper production, causing significant economic losses in different countries, including China, Korea, India, Indonesia, and Thailand [8, 10–13]. In Korea, the *C. acutatum* species complex stands out as the predominant pathogen responsible for the disease, infecting both immature and mature pepper fruits [14]. Specially, *Colletotrichum scovillei* is the major causal agent of anthracnose of chili in Korea [15]. Typical anthracnose symptoms on pepper fruit include sunken necrotic tissues and concentric rings of acervuli, which reduce fruit quality [11]. Anthracnose disease causes pre-and post-harvest pepper fruit rot and reduces their marketability. Various disease management strategies have been implemented to control anthracnose in pepper, including crop management systems (crop rotation, proper soil drainage, and field cleaning of infected plant parts), biological agents, and/or chemicals, and cultivation of resistant genotypes [5, 16]. However, the development of resistant cultivars is the best long-term strategy to control the disease. This is because it reduces the amount of money that is spent on chemical and mechanical methods of disease control [17, 18] and allows a more sustainable crop management.

Genetic analysis studies have provided insights into the inheritance patterns of resistance to *Colletotrichum* species in chili crops, highlighting the importance of species, isolates, and fruit maturation stages [19]. Marker-Assisted Selection (MAS) has emerged as a crucial tool in the chili crop improvement program, enabling the efficient selection of multiple resistance genes for pyramiding into a single genotype [20]. Research findings have revealed varying inheritance patterns for anthracnose resistance in different pepper varieties. For instance, an interspecific cross between “Yeoju” and ‘Daepoong-cho’ demonstrated that resistance against *C. acutatum* is controlled by a monogenic recessive gene [21]. In contrast, genetic analysis of *C. chinense* (PBC932) revealed mostly dominant resistance observed in chromosome P5 for both green and red fruit [22]. Similarly, investigations on the *C. annuum* breeding line ‘83–168’ identified a single dominant gene responsible for resistance against *C. acutatum* [23]. Dominant resistance genes have also been reported in *C. baccatum* PBC80 [24], while differential gene expression at different fruit maturity stages has been observed in *C. chinense* and *C. baccatum* [25]. Understanding the genetic basis of resistance inheritance is crucial for developing effective breeding strategies and selecting resistant cultivars against *Colletotrichum* pathogens in pepper crops.

Several methods have been developed and used to select a core collection with maximum genetic diversity based on morphological characteristics and/or passport information [26]. Approaches such as genotyping-by-sequencing (GBS) are currently being used to genetically characterize germplasm collections based on single nucleotide polymorphisms (SNPs) [27] to develop a unique genetic profile for each accession, allowing analysis of the genetic diversity in the accession collection and the genetic relationships between germplasms [28–30]. Genome-wide association studies (GWAS) provide high resolution through accumulated historical recombination in natural populations, varieties, breeding materials, and collections of landraces [31]. The purpose of a GWAS is to ascertain the associations between genotypes and phenotypes by comparing the allele frequencies of genetic variants between individuals who are ancestrally similar but have phenotypic differences and by analyzing genetic variants across multiple genomes to identify those that are statistically associated with a particular disease or trait [32].

Therefore, this experiment was conducted using a total of 197 *C. chinense* accessions from different countries. These accessions were evaluated for their resistance to anthracnose both in the field and in vitro in different crop years to identify resistant germplasm. The experiment started in 2018 with the *C. acutatum* isolate ‘KSCa-1’. In 2019, a highly virulent and widely distributed *C. scovillei* isolate named ‘Hana’ was identified. Consequently, the studies conducted in 2019 and 2020 utilized the ‘Hana’ isolate. The study aims to identify SNPs associated with anthracnose resistance and the underlying genes through GWAS analysis. The findings from this study will provide valuable insights for future breeding programs, ultimately leading to the development of improved disease-resistant pepper varieties.

## Results

### Evaluation of *C. chinense* germplasm resistance against *anthracnose*

The resistance of 197 pepper (*Capsicum chinense*) accessions deposited in the Republic of Korea’s National Agrobiodiversity Center were evaluated for their response against the virulent pathogen *C. acutatum* isolates ‘KSCa-1’ and *C. scovillei* isolate ‘Hana’ in field and in vitro methods for three consecutive years (2018 to 2020). Multiple genes of the isolate ‘Hana’ were sequenced and its identity as *C. scovillei* was confirmed as presented in the phylogenetic tree (Supplementary Figure S1). The in vitro experiment was carried out to assess *C. chinense* resistance to isolate ‘KSCa-1’ in 2018 and isolate ‘Hana’ in 2020. Disease resistance testing in the field was carried out in 2019 and 2020 against the *C. acutatum* isolate “Hana”. Table 1 summarizes the distribution of germplasm by disease severity score for each year. During the 2018 in vitro experiment with isolate ‘KSCa-1’, the number of germplasms according to the disease scores was as follows: 74 (0–1), 56 (1–2), 30 (2–3), and 37 (3–4), whereas in the 2020 in vitro experiment with isolate “Hana”, the number of germplasms according to the disease score was 48 (0–1), 39 (1–2), 50 (2–3), and 60 (3–4). Regarding the field experiment in 2019, 124 germplasms had a disease score that ranged from 0 to 1, 39 (1–2), 20, (2–3), and 14 (3–4). On the other hand, in the 2020 field experiment, 26 germplasms had a disease score of 0–1, 73 (1–2), 62 (2–3), and 36 (3–4).

**Table 1:** The frequency distribution of *C. chinense* germplasm based on disease score in both in vitro and field inoculation methods from 2018 to 2020

Year	Experiment type	Isolate	Disease severity score				
			0–1	1–2	2–3	3–4	Total
2018	In vitro	KSCa-1	74	56	30	37	197
2019	Field	Hana	124	39	20	14	197
2020	Field	Hana	26	73	62	36	197
2020	In vitro	Hana	48	39	50	60	197

**Fig. 1:**
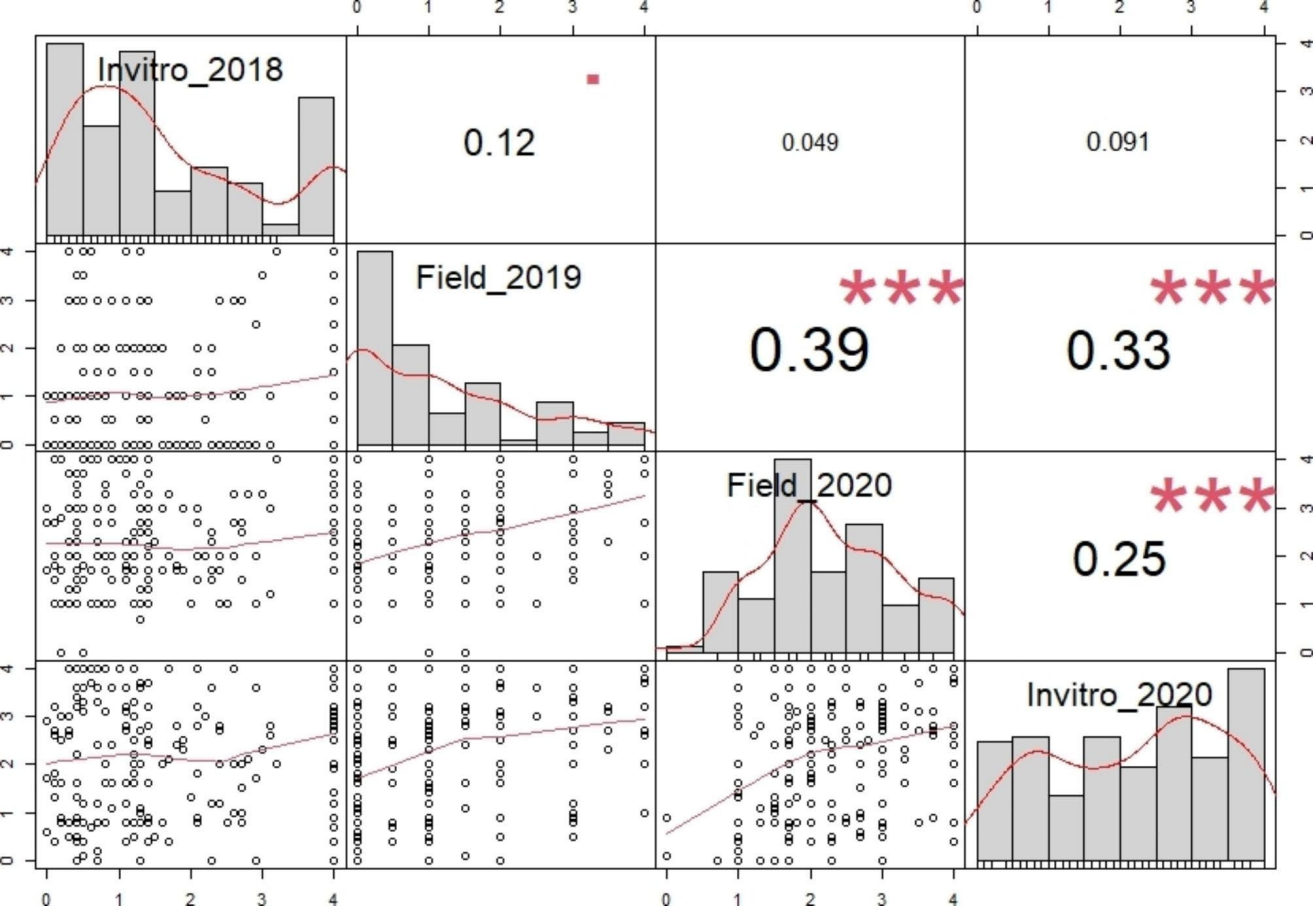
Spearman correlation coefficient between the experimental method and years, based on the anthracnose disease severity scores of the *C. chinense* population. The significance level is represented by asterisks (***), indicating a high level of significance (p < 0.001)

The correlation analysis was conducted based on the disease response of C. *chinense* germplasm between the testing years and inoculation methods (Fig. 1). Regardless of inoculation methods, a positive correlation was observed between testing years. A relatively strong positive correlation was observed between the field inoculation experiments of 2019 and 2020 (r = 0.39***). The correlation between 2018 and 2020 in the in vitro experiment was positive (r = 0.12). The correlation between field and in vitro experiments in 2020 showed a positive correlation (r = 0.25***).

**Fig. 2:**
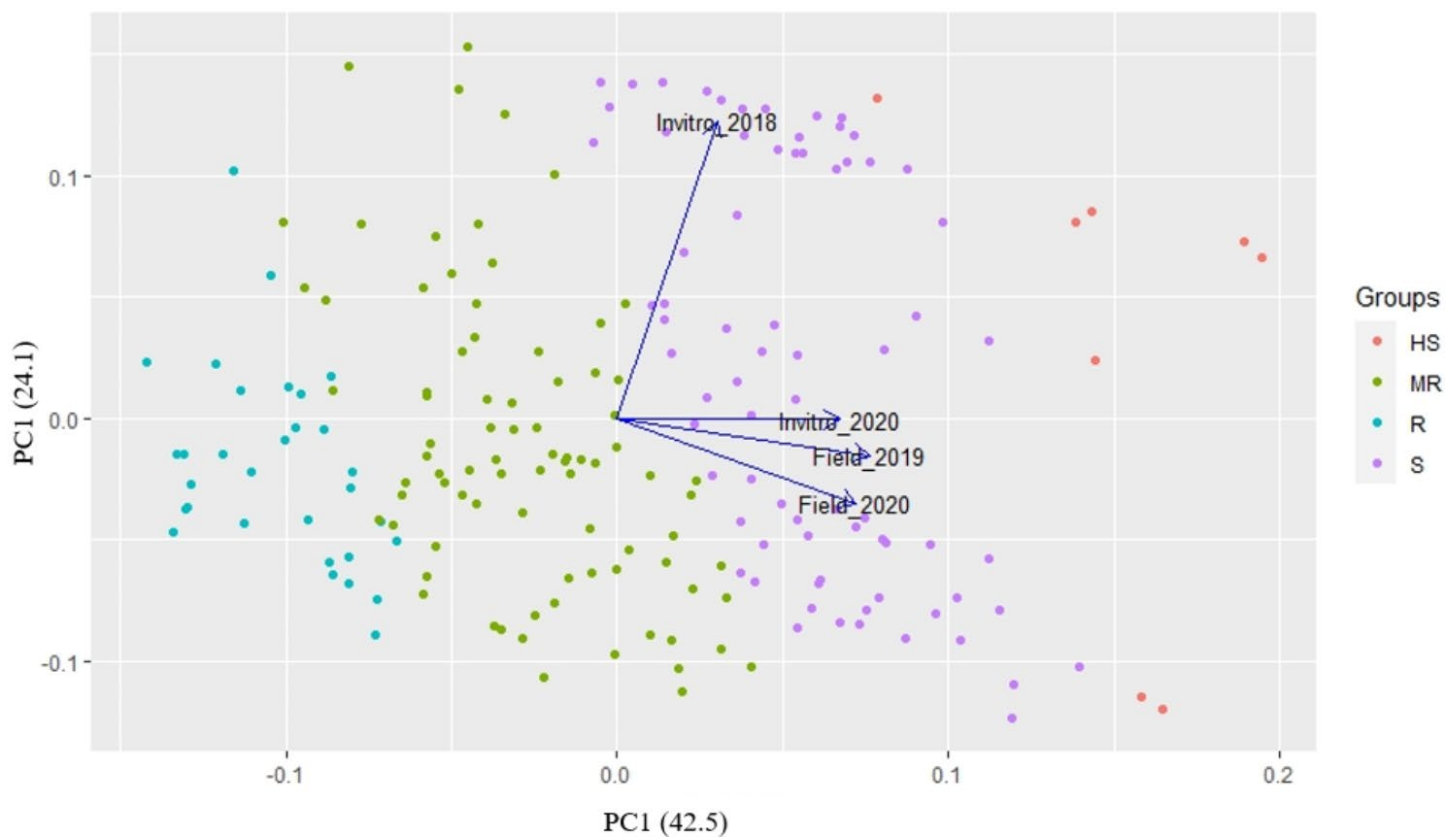
PCA of pepper germplasms based on anthracnose disease severity scores. The color cyan represents accessions classified as resistant (R), green represents moderately resistant (MR), violet represents susceptible (S), and red represents highly susceptible (HS)

A PCA plot was generated using disease data from 197 accessions collected between 2018 and 2020 (Fig. 2). PC1 and PC2 accounted for 66.6% of the total variance, with PC1 alone explaining 42.5% of the variance. The main contributors to PC1 were the field disease data from 2019 to 2020, along with the in vitro disease data from 2020. On the other hand, the primary contributor to PC2 was the in vitro disease data from 2018. The accessions were also grouped based on the average disease score values from all experiment, as shown in Fig. 2. Accessions with disease scores ranging from 0 to 1 (resistant) are represented by the color cyan, those with scores from 1 to 2 (moderately resistant) are represented by green, scores from 2 to 3 (susceptible) are represented by violet, and scores from 3 to 4 (highly susceptible) are represented by the color red.

### Selection of resistant ***C. chinense*** accessions

Six *C. chinense* accessions that showed resistance to anthracnose in all testing years and inoculation methods were selected based on their disease severity score by comparing them with susceptible and resistant controls (Table 2). These six resistant accessions are IT229207, IT236717, IT261436, IT261448, IT284059, and IT305437. Compared to the resistant control (PI 594,137), the selected germplasm showed better resistance to both experimental conditions across the testing years. Of the six resistant genetic resources, five were native to South America (one from Bolivia, one from Brazil, one from Colombia, and two from Peru), and one from Hungary. The resistant control (PI 594,137) had severity scores of 1.4, 1.2, and 2.7 in 2018, 2019, 2020 (field), and 2020 (in vitro), respectively, whereas the susceptible control (Manitta) had a disease score of 4.0 in all testing years regardless of experiment conditions.

**Table 2:** Six selected resistant accessions for anthracnose in both in vitro and field inoculation tests

IT	Accession. Name	Origin	Experiment type (year)			
			In vitro (2018)	Field (2019)	In vitro (2020)	Field (2020)
229,207	chi 16/1028-2	Hungary	0.3	0.0	0.5	1.0
236,717	C04531	Bolivia	0.7	1.0	0.0	1.0
261,436	C 04891	Peru	0.2	1.0	0.9	0.0
261,448	C 04695	Brazil	0.7	0.0	0.8	1.0
284,059	COL NO.399	Peru	0.7	0.0	0.2	1.0
305,437	C 04462	Colombia	0.1	0.0	0.4	1.0
control	Manitta	Korea	4	4	4	4
control	PI 594,137		1.4	1.2	-	2.7

Note: Susceptible control: Manitta, resistant control: PI 594,137

### Genome-wide association analysis

The GBS library was constructed from 197 pepper accession and sequenced using the Illumina Hiseq 2000 platform (Illumina, Madison, WI, USA) and generated approximately 1.2 billion sum of mapped reads with an average mapping depth of 16.96× for a single accession. The summary of GBS statistics of 197 pepper germplasm summarized in Table S1. The SNP matrix was generated and classified as homozygous (SNP read depth ≥ 90%), heterozygous (40% ≤ SNP read depth ≤ 60%) and etc. SNP (20% ≤ read rate < 40%, 60% < read rate < 90%) (Table S2). The SNPs filtering processes are described in Table 3. From the sequences of 197 pepper accession 1,034,421 SNPs were detected with minor alleles frequency (MAF > 5%) covering 12 chromosomes.

**Fig. 3:**
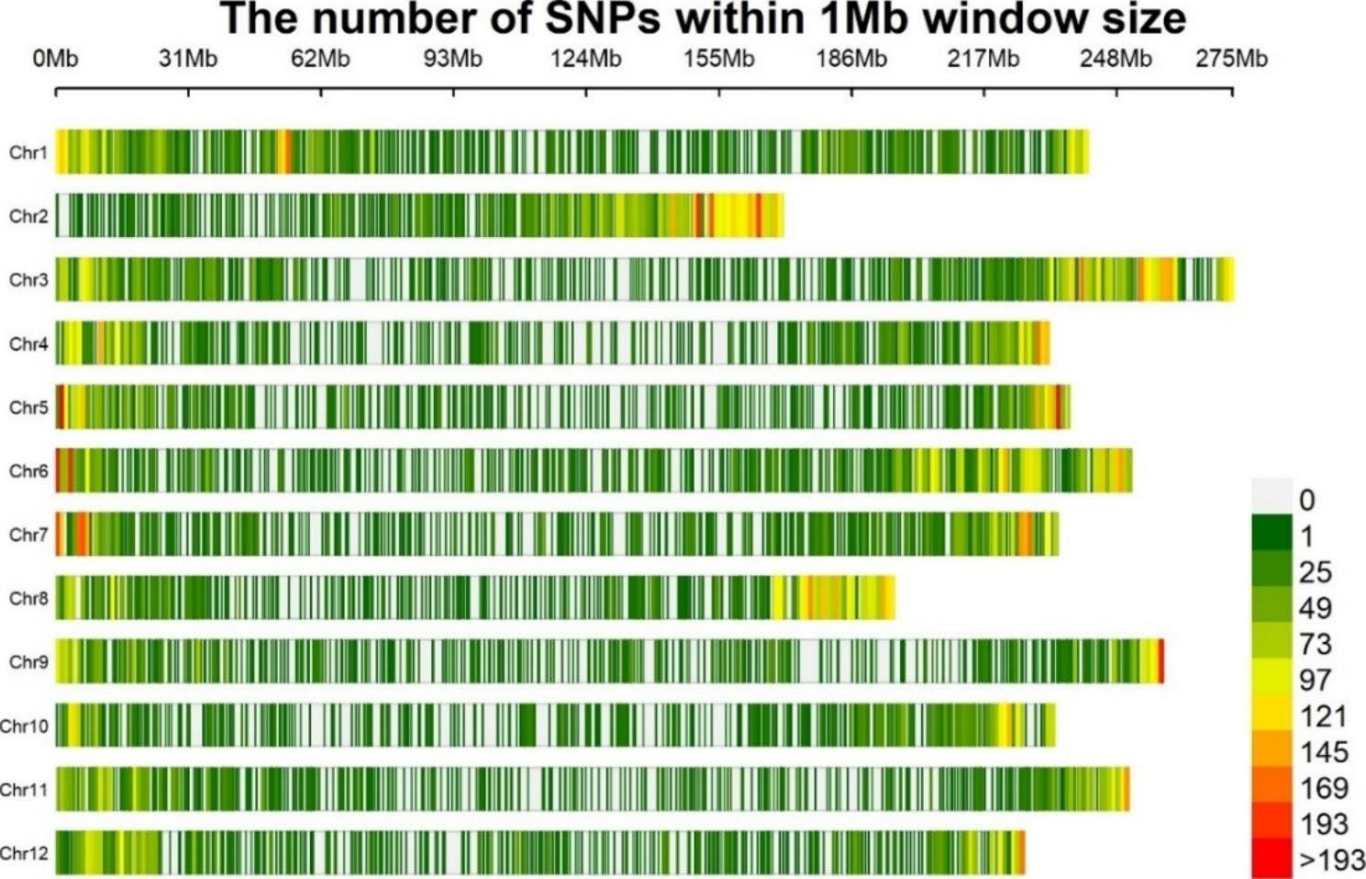
Distribution of 53,518 SNPs across all 12 chromosomes. The figure shows the number of SNPs within 1 Mb window size and reflects the SNP density on each chromosomes used during the present study for GWAS

**Fig. 4:**
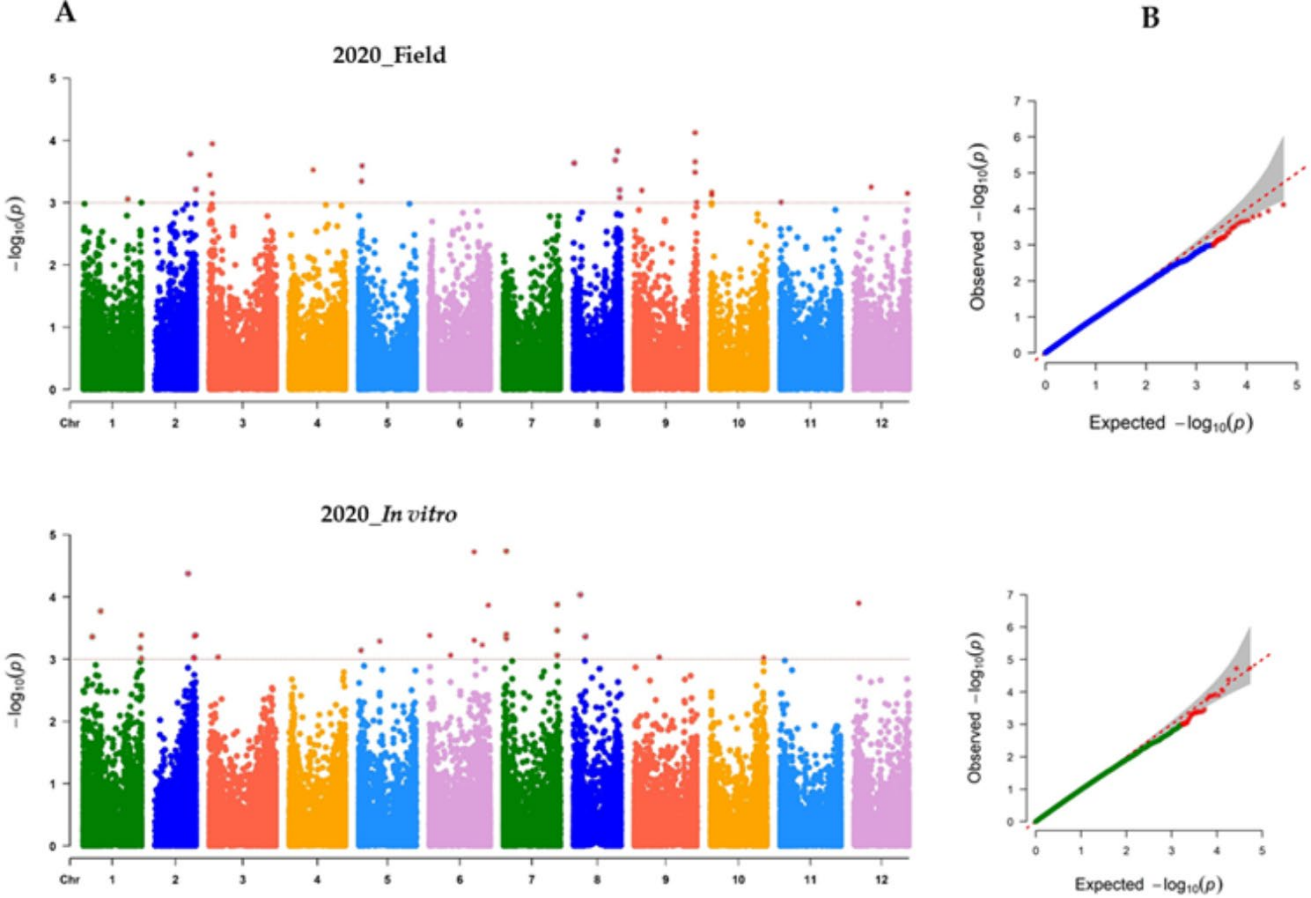
Manhattan (**A**) and quantile–quantile (QQ) (**B**) plots resulting from the genome-wide association study (GWAS) for anthracnose resistance in pepper. Red dotted line indicates the GWAS threshold (p < 0.001)

**Fig. 5:**
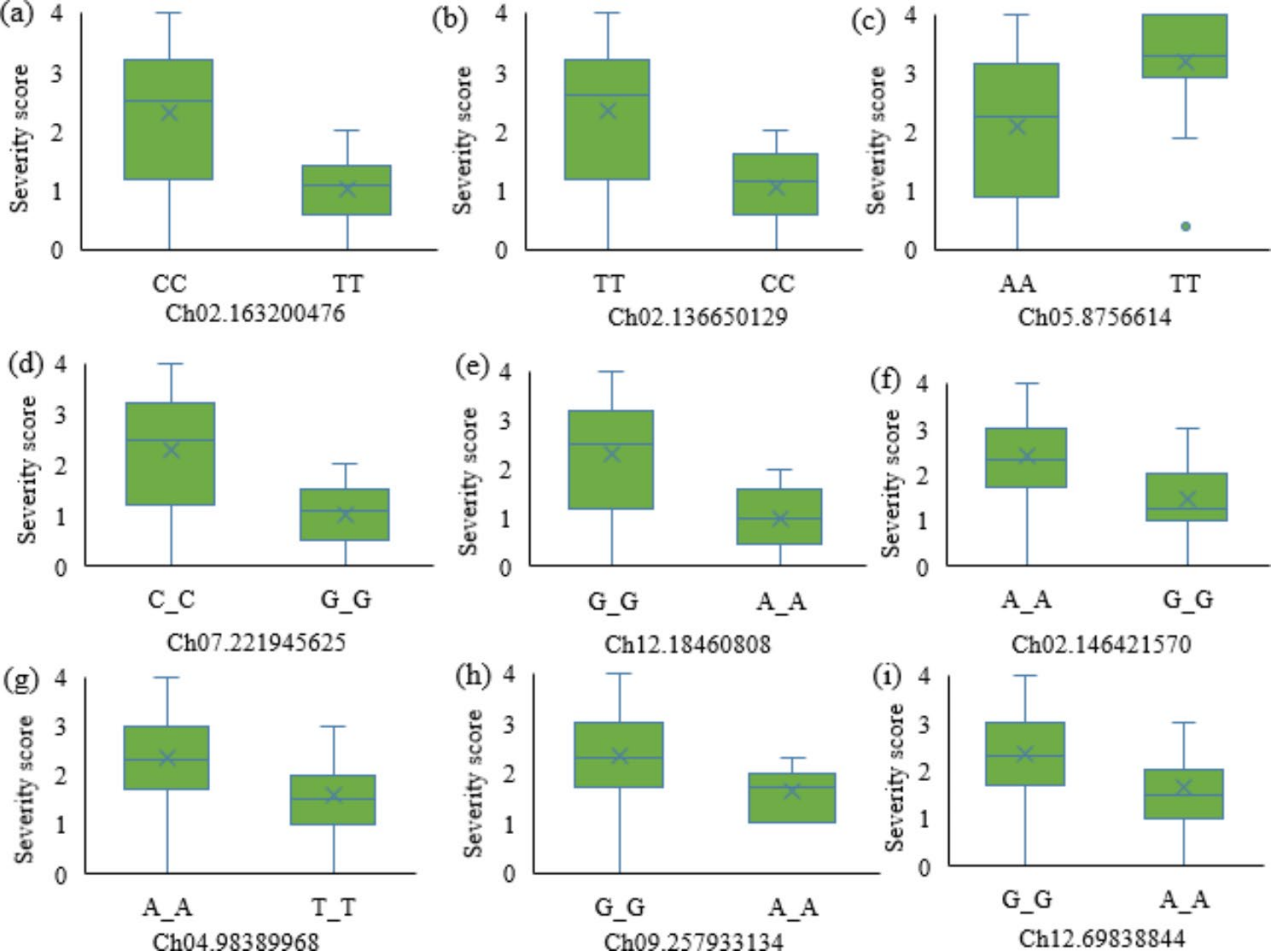
Boxplots depicting the significantly associated SNPs with anthracnose in *C. chinense* based on the in vitro (**a–e**) and field (**f–i**) experiments. The x-axis represents the chromosome number and position (bp), while the y-axis represents the disease severity score. The median is indicated by the horizontal line within the box, and the mean is represented by the ‘x’ symbol

Finally, a total of 53,518 SNPs were obtained after filtering with the combination of MAF (> 5%) and missing data (< 30%). The number of SNPs retained on all 12 chromosomes within a 1 Mb window size is presented in Fig. 3. Genome-wide association analysis was conducted with 53,518 SNPs to identify SNP markers associated with anthracnose resistance. the 2020 field and in vitro experimental disease severity scores was used for GWAS analysis. The association analysis results for both the in vitro and field experiments of 2020 were visualized in Manhattan and QQ plots (Fig. 4A and B). A total of 57 significantly associated SNPs were found in both experimental conditions (Tables 3 and 4). The details of all significantly associated SNPs in both experimental conditions are summarized in Table S3. There were seven significant SNPs on Ch02, Ch06, and Ch02, and six SNPs on Ch01, Ch07, and Ch09. Relatively, the smallest number of significant SNP markers was found on Chr04 and Chr11, with 1 SNP on each chromosome. Based on the GWAS analysis of field and in vitro experiment data, the number of significantly associated SNPs was 25 and 32, respectively. In the GWAS results of the 2020 field experiment, significantly associated SNPS were detected across chromosomes except on Chr06 and Chr07. The number of significantly associated SNPs on Chr09, Chr08, Chr05, and Chr03 were 5, 4, 3 and 3, respectively (Table 3). According to the 2020 in vitro experiment, 14 of the 25 significantly associated SNPs were found in protein-coding genes, while the other 11 were found in the intergenic region. Regarding the 2020 in vitro experiment, the SNPs that were strongly linked were found on all chromosomes except Chr04 and Chr11. The number of SNPS on Chr06, Chr07, Chr01 and Chr02 was 7, 6, 5 and 5, respectively (Table 4). Among the 32 SNPS, 19 were found on protein coding genes and 13 were in the intergenic regions. All six SNPs discovered on Chr07 were in the intergenic region (Table 3). Multiple SNPs were found in the following genes; CA05g03560, CA08g18220, CA10g01780, CA06g12230, and CA12g05320 (Tables 3 and 4). Figure 5 displays box plots of selected SNPs significantly associated with anthracnose based on the *C. chinense* GWAS panels.

**Table 3:** Summary of SNPs filtering process

Filter Stage	Filtering criteria	No. of SNPs
1	Total SNPs	2,170,959
2	MAF (minor allele frequency) > 5% *^1^	1,034,421
3	Missing data < 30% *^2^	182,296
4	Missing data < 30% & MAF > 5%	53,518

(*^1^) MAF (minor allele frequency) > 5%: SNPs with a minor allele frequency greater than 5% are selected from all samples of the locus. (*^2^) Missing data < 30%: SNPs with missing data less than 30% were selected from all samples of the left

**Table 4:** Significant SNPs associated with anthracnose resistance in GWAS analysis of 2020 in vitro experiment

No.	Chr.	Number of SNPs	Physical position (Mb)	Genomic position (10 kb)	Genic/ Intergenic	Gene ID	Description
1	01	1	72.21–73.21	-	Intergenic	-	-
2	01	1	240.04–241.04	-	Intergenic	-	-
3	01	1	37.43–38.43	24288.7–24288.9	Genic	CA01g29280	Receptor serine-threonine protein kinase, putative
4	01	1	236.30–237.30	22051.9–22052.4	Genic	CA01g26910	Hop-interacting protein THI113
5	01	1	241.12–242.12	-	Intergenic		
6	02	1	136.65–137.65	16184.0–16184.3	Genic	CA02g24920	Tetratricopeptide repeat protein, tpr, putative
7	02	1	168.33–169.33	16957.5–16957.7	Genic	CA02g30290	Detected protein of unknown function
8	02	1	163.79–164.79	16542.3–16542.4	Genic	CA02g27150	COBRA protein
9	02	1	163.20–164.20	-	Genic	CA02g26870	E3 ubiquitin-protein ligase UBR4
10	02	1	160.84–161.84	-	Intergenic	-	-
11	03	1	35.50–36.50	3105.6–3106.1	Genic	LOC107864021	Translocase of chloroplast 159
12	05	1	86.18–87.18	14047.0–14047.4	Genic	CA05g11210	Detected protein of unknown function
13	05	1	8.75–9.75	747.5–748.0	Genic	CA05g03080	Mas-binding factor MBF2
14	06	2	184.92–185.92	18812.8–18813.7	Genic	CA06g12230	PREDICTED: protein cornichon homolog 4-like
15	06	1	243.13–244.13	23078.2–23078.4	Genic	CA06g24570	Hop-interacting protein THI033
16	06	1	0.70–1.70	21404.8–21405.6	Genic	CA05g15150	Sodium/hydrogen exchanger
17	06	1	218.56–219.56	21595.1–21596.2	Genic	CA06g17750	Tyrosine kinase family protein isoform 1
18	06	1	86.17–86.67	9372.0–9373.1	Genic	CA06g07190	Protein binding protein, putative
19	06	1	86.85–87.85	9423.8–9424.1	Genic	CA06g07210	Scythe/bat3, putative
20	07	3	10.46–11.46	-	Intergenic	-	-
21	07	3	221.95–222.95	-	Intergenic	-	-
22	08	1	27.60–28.60	23339.6–23339.7	Genic	CA01g28770	Pentatricopeptide repeat protein
23	08	1	48.77–49.77	-	Intergenic	-	-
24	09	1	102.37–103.37	-	Intergenic	-	-
25	10	1	220.27–221.27	-	Intergenic	-	-
26	12	2	8.46–19.46	1667.5–1667.9	Genic	CA12g05320	Galactose oxidase/kelch repeat superfamily protein isoform 2

**Table 5:** Significant SNPs associated with anthracnose resistance in GWAS analysis of 2020 field experiment

No.	Chr.	Number of SNPs	Physical position (Mb)	Genomic region (10 kb)	Genic/ Intergenic	Gene ID	Description
1	01	1	183.73–184.73	-	Intergenic	-	-
2	02	1	145.92–146.92	14895.0–14895.3	Genic	CA02g17350	Serine/threonine-protein kinase
3	02	1	169.27–170.27	-	Intergenic	-	-
4	03	2	10.85–11.85	-	Intergenic	-	-
5	03	1	1.42 − 2.42	1095.4–1095.6	Genic	CA03g04510	CC-NBS-LRR protein, putative
6	04	1	97.89–98.89	-	Intergenic	-	-
7	05	1	12.67–13.67	1126.4–1126.6	Genic	CA05g04030	Detected protein of unknown function
8	05	2	10.21–11.21	929.6–930.3	Genic	CA05g03560	Diphthine-ammonia ligase-like isoform X1
9	08	1	181.65–182.65	13205.0–13206.1	Genic	CA08g12160	ACS2
10	08	1	171.69 − 172.69	-	Intergenic	-	-
11	08	1	1.74–2.74	27032.3–27032.7	Genic	CA01g33750	Nuclear RNA binding protein (Fragment)
12	08	2	190.33–191.33	14289.7–14290.1	Genic	CA08g18220	Putative ternary complex factor MIP1
13	09	3	250.67–251.67	-	Intergenic	-	-
14	09	1	30.11–31.11	2128.2–2128.7	Genic	CA09g04480	Retinol dehydrogenase
15	09	1	257.43–258.43	25163.1–25163.7	Genic	CA09g17980	F-box-containing protein 1
16	10	2	4.69–5.69	383.9–384.5	Genic	CA10g01780	DNA-directed DNA polymerases, putative
17	11	1	3.65–3.65	84.3–84.4	Genic	CA11g00310	Receptor-like protein kinase
18	12	1	69.84–70.34	-	Intergenic	-	-
19	12	1	219.99–220.50	-	Intergenic	-	-

## Discussion

Developing anthracnose-resistant *Capsicum* cultivars relies on identifying resistance sources and understanding the genetic basis, crucial for combating this economically important disease [33]. In the current study, six resistant accessions to *C. acutatum* were found in the *C. chinense* population during field and in vitro disease evaluation methods, and their disease scores fall between 0 and 1 (Table 2). In vitro inoculation of isolate KSCa-1 in 2018 had weak correlations with field and in vitro experiments in 2019 and 2020. This is mainly because the degree of severity in 2018 using isolate KSCa-1 was comparatively low in most of the pepper accessions tested. The PCA analysis effectively demonstrates the distinction between resistant and susceptible accessions, irrespective of experimental type and isolates, based on the overall average disease score values (Fig. 2). Global efforts to enhance anthracnose resistance in *Capsicum* cultivars necessitate evaluating diverse pepper germplasm resources for selecting resistant materials, including previous studies on *capsicum* spp. from different countries [34, 35]. According to the previous studies, a major resistance locus controlled *Capsicum* anthracnose [36], and a single recessive gene was responsible for resistance to *C. capsici* in ‘PBC932’ [21]. Another study showed that the resistance of *C. chinense* “PBC932” against *C. acutatum* was controlled by two complementary dominant genes in green fruits and two recessive genes in red fruits [37].

In this study, a GWAS was conducted using 53,518 SNPs to identify significantly associated SNPs for anthracnose resistance, revealing their presence across all chromosomes, including Ch05, Ch07, Ch10, and Ch12. This widespread distribution highlights the extensive genetic basis of anthracnose resistance in the investigated *Capsicum* species. Moreover, the inheritance analysis of the PBC932 cultivar, known for its resistance to both green and red fruit stages, supported our findings by demonstrating the involvement of well-known genes within the genomic interval on chromosome P5 [19, 24, 25]. These findings provide valuable insights into the major genes or quantitative trait loci (QTL) contributing to anthracnose resistance, particularly in relation to specific pathogens and *Capsicum* species. Additionally, previous studies have reported similar results, identifying several QTLs associated with anthracnose resistance on chromosomes P5, P7, P10, and P12 [19].

An SNP (Ch11: 3,654,665 bp) showed a significant association with anthracnose resistance was detected within a gene responsible for encoding the receptor-like kinase protein. Receptor kinases play a vital role as receptors that bind to molecules involved in signaling pathways [38]. These kinases are members of several gene families that play important roles in extracellular signal recognition and transduction [38]. Plant receptor kinases, for example, are involved in a wide range of physiological and biochemical processes induced by plant hormones and environmental cues, such as self-incompatibility, endosperm and pollen development regulation, flower shedding, response to brassinosteroid and plant disease and environmental stress resistance [39, 40]. Two SNPs significantly associated with anthracnose resistance were identified in Ch01 (37,429,407 bp) based on the in vitro experiment, and in Ch02 (146,421,570 bp) based on field conditions. These SNPs are located within two genes, CA01g29280 and CA02g17350, which encode serine/threonine-protein kinases (Tables 3 and 4). Serine/threonine protein kinases (STKs) are receptor proteins that mediate signal transduction in plant defense responses [38]. STKs are primarily involved in the recognition and transduction of pathogen-derived signals during plant-microbe interactions [38]. A previous study focused on exploring the contribution of four candidate genes that encode STKs to anthracnose resistance in the common bean (*Phaseolus vulgaris* L.) [41]. The study revealed significantly higher expression levels of these genes in the resistant genotype compared to the susceptible genotype. Specifically, Phvul.001G243600 and Phvul.001G243700 genes exhibited approximately 15-fold and 90-fold higher expression levels in the resistant genotype, respectively, even before inoculation [41]. Several STK resistance genes have been cloned from various plant species, including Pto and Prf from tomato [42, 43], Xa21 from rice (Song et al., 1995), RPS5 and PBS1 from Arabidopsis [44, 45], and Rpg5 from barley [46]. Pto encodes a serine/threonine protein kinase that confers resistance to the bacterial pathogen *Pseudomonas syringae* pv. tomato, which expresses the avirulence gene avrPto [42]. The tobacco NrSTK gene encodes a serine/threonine protein kinase with conserved motifs typical of protein kinases, with high sequence similarity to the tomato serine/threonine kinase Pto, which was cloned and studied for resistance to black shank disease [38]. The study concludes that the NrSTK gene, which encodes a serine/threonine protein kinase, functions as a black shank resistance gene in tobacco [38]. The result of our study also strengthens that the serine/threonine-protein kinase gene has a role in anthracnose resistance, as mentioned in previous studies about its involvement in disease resistance. Protein phosphorylation/dephosphorylation are important signaling events in plants that are triggered by biotic and abiotic stress, as well as developmental pathways [47–49]. Serine/threonine protein kinases (EC 2.7.11.1) are host immune receptors that have been shown to play a role in effector-triggered immunity (ETI) in plants by phosphorylating the OH group of serine or threonine residues, resulting in a functional change in the target protein [50, 51]. The critical elucidation of specific protein kinases’ physiological roles in plants, under abiotic and biotic stress, is essential for comprehending and identifying key players in defense pathways. Furthermore, protein kinases are regarded as suitable targets for gene modification, contributing to crop improvement [52, 53].

Disease-resistant markers are primarily associated with resistance (R) proteins, the majority of which feature a domain comprising nucleotide-binding sites and carboxy-terminal leucine-rich repeat regions (NBS-LRR) [54–58]. The presence of NBS-LRR genes has been identified in numerous plant species, leading to significant advancements in disease resistance research [58]. A SNP (Ch03, 1,918,096 bp) was found in a gene that encodes CC-NBS-LRR protein (Table 3). Based on deduced N-terminal structural features, NBS-LRR can be classified into two subfamily groups: toll/interleukin-1 receptor (TIR-NBS-LRR; TNL) and coiled-coil domain (CC-NBS-LRR; CNL) [59, 60]. The disease resistance protein, which is resistance to *P. syringae* 5 (RPS5) in Arabidopsis thaliana, is a well-characterized CNL protein that confers resistance to *Pseudomonas* pv. tomato (Pto) strain DC3000 carrying the heterologous avirulence gene avrPphB [44, 61, 62].

The results of the BLAST searches indicate that the SNP marker on chromosome 02 (specifically, in the region between positions 163.20 and 164.20) corresponds to a gene annotated as an E3 ubiquitin ligase protein. Additionally, a similar finding was reported for a significantly associated SNP marker for stripe rust in wheat on chromosome 6AL (Tdurum_contig29607_413), which also encodes an E3 ubiquitin ligase protein [63]. The study by You et al. [64] suggests that E3 ubiquitin ligase proteins are important modules in plants for regulating innate immunity and programmed cell death. These proteins significantly contribute to enhancing antimicrobial defense mechanisms while preventing the occurrence of autoimmunity. Furthermore, the study indicates that E3 ubiquitin ligases are involved in broad-spectrum disease resistance, implying that they play a role in protecting plants against a wide range of pathogens. However, it is important to note that the specific functions and mechanisms of E3 ubiquitin ligases can vary among different genes and species. Therefore, further research would be necessary to determine the exact role and significance of these E3 ubiquitin ligase proteins in the context of the SNP markers found on chromosome 02 in the current study with respect to their function in anthracnose resistance and on chromosome 6AL with respect to their function in stripe rust resistance in wheat [64]. A significant SNP was found on Ch05 in a gene that encodes a putative pentatricopeptide repeat-containing protein (PRP). A study demonstrated that a PRP protein was involved in disease resistance and salt tolerance in rice [65]. While PPR genes are primarily involved in post-transcriptional regulation of gene expression in mitochondria and chloroplasts, they have been found to share common features with disease resistance genes (R genes) which are known for their role in plant defense against pathogens [66]. Further studies are essential to understand the functions of these genes in which the selected SNPs are found and their association with disease resistance.

## Conclusion

This study evaluated pepper accessions collected from different countries for resistance to *C. acutatum* isolate ‘KSCa-1’ and *C. scovillei* isolate ‘Hana’ that cause anthracnose on *capsicum* spp. A phenotypic evaluation of *C. chinense* against anthracnose was carried out, and six resistant accessions were identified. These accessions showed better resistance compared to the resistant control pepper (PI 594,137). Based on the results of this experiment, the selected resistant materials can be used as a potential source of anthracnose resistance for pepper breeding and genetic studies. Using GWAS, SNPs associated with anthracnose resistance on different chromosomes and their positions in candidate genes were identified. Furthermore, the identified SNPs can be further investigated as potential markers for resistance screening of *C. chinense* genetic resources. As mentioned in several studies, genes that encode protein kinase receptors have been known as a source of resistance for several diseases. The presence of significantly associated SNPs in this study in a gene that encodes a protein kinase receptor, including serine/threonine-protein kinase, can be an indication of its involvement in anthracnose resistance development in *Capsicum* spp.

## Materials and methods

### Plant materials and pathogen (*Colletotrichum acutatum and Colletotrichum scovillei*)

In this study, 197 pepper germplasms from *Capsicum chinense* collected from different countries were used. This experiment was conducted for three consecutive years from 2018 to 2020. In 2018, ten plants from each accession were grown in greenhouses at the National Agrobiodiversity Center in Jeonju, and for two years from 2019 to 2020 at Hana Seed Co.‘s experimental sites in Ansang, Korea. This experiment was triplicated. Resistant control (PI 594,137) and susceptible control (Manitta) pepper germplasms were used. The pepper plants were grown according to the standard pepper cultivation methods of the Rural Development Administration (RDA, Jeonju, Korea) [67]. The field performances of the pepper accessions against *C. acutatum* and *C. scovillei* isolates were evaluated. The introduction number (IT) and geographic origin of the 197 *C. chinense* accessions used in this study are presented in Supplementary Table S4.

The single spore isolation was performed following the protocol described by Oo et al. [15] with minor modifications. The diseased fruits displaying characteristic symptoms were cut into small 5 mm fragments and surface sterilized using a 1% sodium hypochlorite (NaOCl) solution for 3 min. After three rinses with sterilized distilled water and drying on sterilized tissue paper [68], the prepared fruit fragments were placed on Petri dishes and incubated in a dark-light chamber at 25 ± 2 °C with a 12-hour light-dark cycle. Following a 2-day incubation period, spore layers were isolated using autoclaved toothpicks or glass sticks and mixed with distilled water in a tube. The spore-water mixture was then streaked onto Water agar media and allowed to grow for 3 days at 25 ± 2 °C [69]. Finally, a single isolated spore from the emerging fungus was transferred onto a potato dextrose agar (PDA) plate to establish a pure culture.

The ‘KSCa-1’ isolate has been identified as *C. acutatum*, whereas the ‘Hana’ isolate is classified as *C. scovillei* (Supplementary Figure S1). *C. scovillei* is within the *C. acutatum* species complex, indicating a close relationship between the two species [70]. Two isolates from *C. acutatum* and *C. scovillei* were used to evaluate the resistance of pepper accessions in different years of cultivation. In 2018, the ‘KSCa-1’ isolate was used for in vitro evaluation, and the isolate ‘Hana’ in 2019 (field experiment) and 2020 (field and in vitro experiments). Dr. Lee [36] kindly offered us the pathogenic fungus *C. accutatum* (KSCa-1 isolate). Isolate ‘Hana’ was identified from the pepper cultivation fields of Hana Seed Co. (Anseong, Korea) (Supplementary Figure S1).

### Inoculum preparation

The inoculum preparation was performed according to the procedure of Kim et al. [34]. Potato dextrose agar (PDA) plates (Sigma Chemical Co., St. Louis, MO, USA) were used to grow the isolates at 28 °C under 16 h fluorescent light/ 8 h dark in a temperature-controlled incubation chamber. After the isolates had grown for seven days, the PDA plates were flooded with distilled water, and the fungal culture was gently scraped off the plates. A hemocytometer was used to adjust the inoculum to a density of 1.0 × 10^5^ conidia mL^− 1^.

### Inoculation methods

In both in vitro and field experiments, inoculation was performed. In vitro experiments were carried out with the *C. acutatum* isolate ‘KSCa-1’ in 2018 and the *C. scovillei* isolate ‘Hana’ in 2020. Well-developed pepper fruits were harvested from pepper plants. The in vitro inoculation was performed according to the protocol described by Kim et al. [71] with minor modifications. The fruits were treated with 10% Clorox for 3 min, washed in sterile distilled water several times, and dried with sterile paper towels. We placed 10 fruits per accession in resealable plastic bags (25 × 30 cm) containing wet paper towels. The fruit surface was sprayed with inoculum adjusted to 1.0 × 10^5^ conidia mL^− 1^ concentration. To induce disease, the inoculated peppers were immediately sealed to maintain moisture and placed in a growth chamber at 28 °C. After two days of incubation, the resealable plastic bags were opened for two hours at room temperature to prevent excessive moisture from forming on the fruits. Sterile distilled water instead of the conidial suspension was used as control. The incubation period lasted 14 days under the same conditions. This experiment was conducted in triplicate.

Field inoculation with the isolate ‘Hana’ was conducted in the experimental field of Hana Seed Co. located in Anseong, Korea. The isolate ‘Hana’ inoculum concentration used for the field inoculation was 1.0 × 10^5^ conidia mL^− 1^. To introduce the pathogen into the field, the inoculum was diluted, and a spray inoculation method, similar to pesticide spray application, was employed to ensure widespread distribution and effective dissemination of the pathogen. The initial inoculation carried out around 110 days after planting and was conducted three times prior to the start of the monsoon season. In 2019, the spray inoculations took place on June 27, July 11, and July 24. Similarly, in 2020, the inoculations were carried out on June 27, July 3, and July 9, ensuring a sufficient exposure of the plants to the pathogen.

### Disease severity assessment

In an in vitro experiment, the percentage of infected sites was calculated to evaluate the disease severity with an average of 14 days after inoculation. The disease severity of field inoculation was scored at 4 weeks post-inoculation. The disease severity in both experimental conditions was scored according to the method described by Ro et al. [35]. The scoring system used a range of 0 to 4 scales: 0 indicated no symptoms, 1 indicated symptoms with less than 10% disease incidence, 2 indicated symptoms with 11–20% incidence, 3 indicated symptoms with 21–40% incidence, and 4 indicated symptoms with 41–100% incidence. Supplementary Figure S2 illustrates the disease severity score in both experimental conditions. This scoring was applied to both non-wound inoculated and field-inoculated pepper accessions. The phenotypes were categorized based on their mean disease severity scores: those with a score of 0–1 were considered resistant (R), scores of 1–2 were classified as moderately resistant (MR), scores of 2–3 were categorized as susceptible (S), and scores of 3–4 were classified as highly susceptible (HS).

### Genotyping and genome-wide association analysis

Genomic DNA was extracted using the CTAB method [72] from young leaves of each accession. The amount of DNA is quantified using the standard procedure of the Quant-iT PicoGreen dsDNA Assay Kit (Molecular Probes, Eugene, OR, USA) with the Synergy HTX Multi-Mode Reader (Biotek, Winooski, TV, USA) and normalized to 12.5 ng µL^− 1^. DNA was digested with ApeKI restriction enzyme (New England Biolab) at 75 °C for 3 h. Libraries for GBS were constructed according to previously described protocols [27, 73] with minor modifications. The GBS libraries were sequenced using Illumina Hiseq 2000 platform (Illumina, Madison, WI, USA) with 151 bp of paired-end reads. The cleaned reads generated after passing the pre-processing process were mapped to *C. chinense* reference genome v1.2 (http://peppergenome.snu.ac.kr/).

Barcode sequence was used for demultiplexing, followed by adapter sequence removal and sequence quality trimming. Adapter and barcode sequences were eliminated using the software Cutadapt (version 1.8.3) [74]. DynamicTrim and LengthSort programs of the SolexaQA (v.1.13) package [75] were used to remove low-quality sequences. For DynamicTrim, a phred score ≥ 20 was applied. For LengthSort, a short read length of ≥ 25 pb was applied. BWA (BurrowsWheeler Aligner, ver.0.6.1-r104) [76] generated cleaned reads, passed the preprocessing process, and performed mapping to the *C. chinense* reference genome v1.2 (http://peppergenome.snu.ac.kr/). A SAM file was created, and default values were used, except for the following options: a seed length (− l) of 30, maximum differences in the seed (− k) of 1, number of threads (− t) of 16, mismatch penalty (− M) of 6, gap opening penalty (− O) of 15, and gap extension penalty (-E) of 8.

The clean reads were mapped to the reference genome sequence. The resulting SAM files were utilized for raw SNP discovery using SAMtools (0.1.16) [77], and consensus sequences were extracted from the obtained data. SNP validation was conducted using SEEDERS in-house script [78] before SNP detection; raw SNP detection was performed, and default values were used except for the following options: a minimum mapping quality for SNPs (− Q) of 30, minimum mapping quality for gaps (− q) of 15, minimum read depth (− d) of 3, minimum InDel score for nearby SNP filtering (− G) of 30, SNPs within INT bp around a gap to be filtered (− w) of 15, window size for filtering dense SNPs (− W) of 30, and maximum read depth (− D) of 165.

The SNP matrix was generated by removing the incorrectly identified SNP sites using SNP comparison between samples. SNPs were classified as homozygous (SNP read depth ≥ 90%) or heterozygous (40% ≤ SNP read depth ≤ 60%) and other SNPs, which did not meet the criteria for Homozygous or Heterozygous (Table S2). Finally, a total of 53,518 high-quality SNPs were generated for association analysis. Based on the location information of the reference genome sequence (*C. chinense*. v1.2, http://peppergenome.snu.ac.kr/).

Based on the response of pepper accessions to anthracnose disease across testing years, the 2020 field and in vitro experiments were deemed relatively representative for GWAS analysis, as disease severity was greater in 2020 than in other years. The association analysis was conducted using a dataset consisting of 53,518 SNPs. This analysis utilized the genomic association and prediction integrated tool (GAPIT3) package [79], which is integrated within the R statistical software program (version 4.0.2). The analysis employed a mixed linear model (MLM) [80]. The threshold value was set at p < 0.001 (-log (p) > 3.0) for declaring significant marker-trait association [81].

For the identification of candidate genes associated with the SNP of interest, we conducted a BLAST search using the *Capsicum* genome database (http://peppergenome.snu.ac.kr, *C. annum*.v.1.55) and the NCBI (National Center for Biotechnology Information) database. Our search focused on a 200 kb region surrounding the SNP, including 100 kb on each side. The flanking sequences of the SNP were obtained from the *Capsicum chinense* genome database. Subsequently, we compared these sequences against the *Capsicum* genome database and the NCBI database to identify genes or gene regions that exhibited similarity or alignment. This comprehensive approach allowed for the efficient identification of potential candidate genes associated with the SNP of interest. We reported the physical location of the SNPs within the reference genome of *C. chinense* using a 1 Mb window size. Additionally, we predicted the gene positions based on the *C. annum* reference and the NCBI reference genome regions.

### Statistical analysis

The disease assessment was summarized using Microsoft Excel. Pearson’s correlation between experiment methods and experiment years, as well as principal component analysis (PCA), were carried out using the R statistical software program (4.0.2 version).

### Electronic supplementary material

Below is the link to the electronic supplementary material.

Supplementary Material 1

## Data Availability

The datasets used and/or analyzed during the current study are available in the supplementary file, and additional datasets can be provided by the corresponding author on request. The SNPs data used for GWAS analysis submitted to National Agricultural Biotechnology Information Center (https://nabic.rda.go.kr/nolog/NV-0771-000001/snpVcfView.do) with the following accession number NV-0771.
